# Chromatin Regulator SMARCA4 Is Essential for MHV-Induced Inflammatory Cell Death, PANoptosis

**DOI:** 10.3390/v16081261

**Published:** 2024-08-06

**Authors:** R. K. Subbarao Malireddi, Thirumala-Devi Kanneganti

**Affiliations:** Department of Immunology, St. Jude Children’s Research Hospital, Memphis, TN 38105, USA; malireddi.subbarao@stjude.org

**Keywords:** innate immunity, cell death, coronavirus, murine hepatitis virus, β-coronavirus, gasdermin D, caspase-1, caspase-8, caspase-7, MLKL, PANoptosis, PANoptosome, inflammasome, SMARCA4, CRISPR, inflammation, macrophage

## Abstract

The innate immune system serves as the first line of defense against β-coronaviruses (β-CoVs), a family of viruses that includes SARS-CoV-2. Viral sensing via pattern recognition receptors triggers inflammation and cell death, which are essential components of the innate immune response that facilitate viral clearance. However, excessive activation of the innate immune system and inflammatory cell death can result in uncontrolled release of proinflammatory cytokines, resulting in cytokine storm and pathology. PANoptosis, innate immune, inflammatory cell death initiated by innate immune sensors and driven by caspases and RIPKs through PANoptosome complexes, has been implicated in the pathology of viral infections. Therefore, understanding the molecular mechanisms regulating PANoptosis in response to β-CoV infection is critical for identifying new therapeutic targets that can mitigate disease severity. In the current study, we analyzed findings from a cell death-based CRISPR screen with archetypal β-CoV mouse hepatitis virus (MHV) as the trigger to characterize host molecules required for inflammatory cell death. As a result, we identified SMARCA4, a chromatin regulator, as a putative host factor required for PANoptosis in response to MHV. Furthermore, we observed that gRNA-mediated deletion of *Smarca4* inhibited MHV-induced PANoptotic cell death in macrophages. These findings have potential translational and clinical implications for the advancement of treatment strategies for β-CoVs and other infections.

## 1. Introduction

The innate immune system serves as the first line of defense against infections, using pattern recognition receptors (PRRs) to detect pathogens. In the context of β-coronavirus (β-CoV) infections, such as SARS-CoV-2, the causative agent of coronavirus disease 2019 (COVID-19), these genetically encoded PRRs activate the innate immune response, which can initiate inflammatory responses and cell death [1,2,3]. The innate immune system is a key factor in defining the severity of β-CoV and SARS-CoV-2 infections [4,5,6,7]. While innate immune-mediated cell death is meant to provide host protection and impede viral replication, uncontrolled cell death can result in disrupted immunological responses, cytokine storm, organ damage, and mortality [8]. One particular inflammatory cell death pathway, PANoptosis, has been widely implicated in the pathophysiology of β-CoVs and SARS-CoV-2 [9,10,11,12]. PANoptosis is an innate immune, inflammatory cell death pathway initiated by innate immune sensors and driven by caspases and receptor-interacting protein (RIP) kinases (RIPKs) via PANoptosomes. PANoptosomes are multiprotein complexes assembled by innate immune sensor(s) in response to pathogens, pathogen- or damage-associated molecular patterns (PAMPs or DAMPs), cytokines, or homeostatic changes that drive PANoptosis [13,14,15]. In the context of SARS-CoV-2, PANoptosis can result in cytokine storm and multiorgan damage [9,10,11]. Patients with severe COVID-19 present with increased levels of TNF and IFN-γ, which can drive PANoptosis [9,10]. Additionally, the lethality of IFN therapy in murine β-CoV infection models is driven by PANoptosis [12], suggesting that IFN-mediated pathology during SARS-CoV-2 infection occurs through PANoptosis. Furthermore, a recent RNA-seq dataset analysis established a strong association between PANoptosis and COVID-19 severity [11]. In addition to β-CoV pathophysiology, PANoptosis is linked to several other infections, inflammatory diseases, and cancers [15,16,17,18,19,20,21]. Despite the established role of PANoptosis in β-CoV, and specifically SARS-CoV-2, pathogenesis, the mechanistic understanding of how β-CoVs induce PANoptosis remains incomplete.

To define the molecular mechanisms underlying innate immune inflammatory PANoptotic cell death in β-CoV infections, we investigated putative regulators identified in a prior genome-wide host CRISPR knockout screen for cell death molecules that used MHV as a trigger [22]. MHV is a prototypical murine β-CoV that is commonly used as a model due to its ability to infect organs, induce acute and chronic illness, and promote PANoptosis in bone marrow-derived macrophages (BMDMs) [2,23,24], mimicking several key aspects of human β-CoV biology. Here, we re-evaluated the MHV-induced cell death CRISPR screen and identified the chromatin regulator SWI/SNF-related, matrix-associated, actin-dependent regulator of chromatin, subfamily a, member 4 (SMARCA4) as a critical regulator. Moreover, the loss of *Smarca4* led to a reduction in the cell death and decreased the activation of PANoptosis molecules, suggesting that SMARCA4 plays a critical role in β-CoV-mediated PANoptosis.

Overall, our work highlights the power of using genome-wide screens in macrophage cell lines to identify critical host components involved in innate immune responses. Moreover, addressing the molecular mechanisms that lead to inflammatory cell death in β-CoV infections offers valuable knowledge for identifying potential therapeutic targets in infections.

## 2. Materials and Methods

### 2.1. Mouse Hepatitis Virus (MHV) Culture

The mouse hepatitis virus (A59 strain) was propagated in 17Cl-1 cells as previously described [25]. The viral titer was measured using a plaque assay in 17Cl-1 cells.

### 2.2. CRISPR Screen Analysis

The data generated previously from the MHV CRISPR screen in Brie library-containing immortalized BMDMs (iBMDMs) were used for the analyses, following the same procedures described previously (BioProject: PRJNA1009133) [22]. Briefly, calc_auc_v1.1.py (https://github.com/mhegde/, accessed on 21 September 2020) and count_spacers.py [26] were used to validate and verify the presence of gRNA, followed by the analysis of CRISPR KO screens using MAGeCK-VISPR (version 0.5.7) [27]. The MAGeCK pipeline was used to estimate the log2 (fold change) with significance levels for each gene in the CRISPR screen. The CRISPR screen leveraged the intrinsic MHV-mediated cell death to eliminate susceptible cells, leaving behind only those in which gene deletion protected from MHV-induced cell death. Therefore, genes with a positive fold change were expected to be required for cell death. The enriched genes that were significant in the CRISPR screen were visualized using a volcano plot and an RRA score plot using MAGeCKFlute v2.0.0 [28]. The expression profiles of individual gRNAs for the control and treated samples were visualized using a scatter plot. 

### 2.3. Cas9-iBMDM Culture and Infection

For in vitro validation studies, Cas9-expressing iBMDMs were simultaneously electroporated with two gRNAs targeting *Smarca4* (*Smarca4*-RNA1: CCCTGTGAAGGTGATCCACG, *Smarca4*-RNA2: GAGGTATGTGATGAGCGCGA); the resulting pool of cells was referred to as the *Smarca4-g* sample. The gRNA-electroporated cells were allowed to rest for 5 days to achieve CRISPR-based deletion of the targeted gene. The cells were then expanded and seeded in 12- or 24-well plates at a seeding density of 1 × 10^6^ cells/well and 0.5 × 10^6^ cells/well, respectively, in complete DMEM (DMEM supplemented with 10% heat-inactivated fetal bovine serum (HI-FBS; S1620, Biowest, Lakewood Ranch, FL, USA) and 1% penicillin-streptomycin (15070-063, Thermo Fisher Scientific, Waltham, MA, USA), and rested for approximately 24 h before the experiments. The cells were then washed with PBS and infected with MHV at an MOI of 0.1 in DMEM plain media (Sigma, D6171, St. Louis, MI, USA) with 1% penicillin/streptomycin. After 1 h of incubation with MHV, the cells were supplemented with HI-FBS to a final concentration of 10% and used for IncuCyte imaging or incubated for the indicated times before collecting samples for downstream analyses. 

### 2.4. Cell Death Analysis

The IncuCyte^®^ S3 and/or SX5 Live-Cell Analysis System was used to image and analyze cell death in real time, as previously described [29]. In brief, the control and Cas9-iBMDMs electroporated with *Smarca4* gRNA were seeded in 24-well plates (0.5 × 10^6^ cells/well) and infected with MHV as described above. After 1 h of incubation, propidium iodide (PI; Life Technologies, P3566, Carlsbad, CA, USA) was added to the cells together with HI-FBS. The plate was scanned for fluorescent and phase-contrast images (4 image fields/well) in real time for a total of 18 h at intervals of 1 or 2 h. Cell death was quantified as the percentage of PI^+^ cells. 

### 2.5. Western Blotting

To blot for caspases, Cas9-iBMDMs were lysed along with the supernatant using 125 μL of 4× SDS loading buffer containing 50 μL of caspase lysis buffer (1× protease inhibitors, 1× phosphatase inhibitors, 10% NP-40, and 25 mM DTT). To detect other proteins in the cell lysates alone, the supernatants were aspirated, and the cells were lysed using RIPA buffer. Caspase and RIPA lysates were separated on 10–12% polyacrylamide gels and then transferred onto PVDF membranes. After blocking the membranes with 5% skim milk for 1 h at room temperature, the blots were incubated with their respective primary antibodies overnight at 4 °C. Subsequently, the blots were washed with 1× TBST and incubated with secondary antibodies conjugated with horseradish peroxidase (HRP) for 1 h at room temperature. The GE Amersham Imager 600 was used to develop the blots. In certain cases, the immunoblots were stripped using stripping buffer (Restore Western Blot Stripping Buffer, 21059, Thermo Fisher Scientific) and reprobed for a different protein of interest. The antibodies used were as follows: anti–caspase-1 (AdipoGen, AG-20B-0042, 1:1000, San Diego, CA, USA), anti–caspase-3 (#9662, Cell Signaling Technology [CST], 1:1000, Danvers, MA, USA), anti-cleaved caspase-3 (#9661, CST, 1:1000), anti–caspase-8 (CST, #4927, 1:1000), anti-cleaved caspase-8 (CST, #8592, 1:1000), anti-GSDMD (Abcam, ab209845, 1:1000, Waltham, MA, USA), anti-pMLKL (CST, #37333, 1:1000), anti-tMLKL (Abcepta, AP14272B, 1:1000, San Diego, CA, USA), anti-β-actin (66009–1-IG, Proteintech, 1:5000, Rosemont, IL, USA), and HRP-conjugated secondary antibodies (Jackson ImmunoResearch Laboratories, anti-rabbit [111-035-047], 1:5000; anti-mouse [315-035-047], 1:5000). 

### 2.6. Quantitative PCR

Total RNA was isolated using TRIzol^®^ reagent (Ambion, Waltham, MA, USA, #15596018). A total of 500 ng of RNA was reverse transcribed to cDNA using a high-capacity cDNA synthesis reverse transcription kit (Applied Biosystems, Waltham, MA, USA, #4368813), as per the manufacturer’s instructions. cDNA was used to measure *Smarca4* gene expression in Cas9-expressing iBMDMs with or without *Smarca4* depletion by gRNA treatment (described above). mRNA levels were quantified in real time using Sybr^®^ Green (Applied Biosystems, #4367659) and the Quant Studio™ 7 Flex Real-Time PCR System (Applied Biosystems). The expression levels were normalized to *Actb* for host cellular *Smarca4* gene expression and are presented as % *Smarca4* expression. Forward and reverse primer sequences used in the study were as follows: *Smarca4*, Forward: 5-CAAAGACAAGCATATCCTAGCCA-3, Reverse: 5-CACGTAGTGTGTGTTAAGGACC-3; and *Actb*, Forward: 5-CAGCTTCTTTGCAGCTCCTT-3, Reverse: 5-CACGATGGAGGGGAATACAG-3.

### 2.7. Statistical Analysis

Data analysis was performed using the GraphPad Prism 10.0 software. Data are presented as the mean ± SEM of two or three independent repeats. The Student’s *t*-test was used to determine statistical significance. *p* values < 0.05 were considered significant, where ** *p* < 0.01, *** *p* < 0.001, and **** *p* < 0.0001.

## 3. Results

### 3.1. A Genome-Wide CRISPR Screen for Regulators of MHV-Induced Cell Death Identifies Critical Host Genes in β-CoV Infection

**Figure 1:**
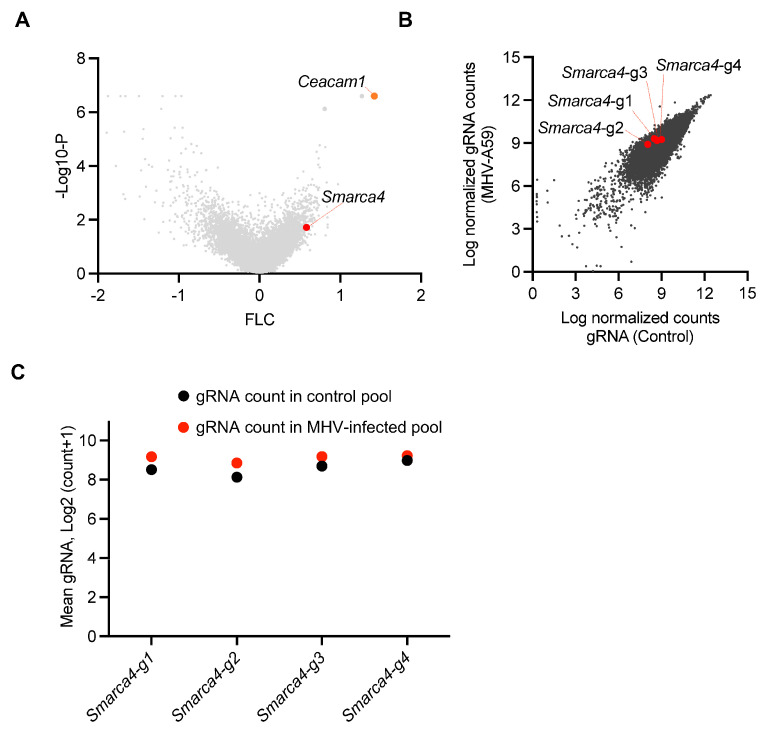
**CRISPR screen identifies host factors that facilitate β-CoV-induced cell death.** (**A**) Volcano plot depicting the log2 mean fold change (FLC) for the gRNAs in the CRISPR screen based on an earlier genome-wide CRISPR screen in immortalized bone marrow-derived macrophages (iBMDMs) infected with mouse hepatitis virus (MHV; MOI 0.1) for 24 h. The newly identified enriched host gene *Smarca4* is labeled alongside the previously identified gene *Ceacam1*. (**B**) Scatter plot illustrating the enrichment of all four gRNAs targeting *Smarca4* in the pool of iBMDMs carrying gRNAs from the whole genome CRISPR screen following MHV infection. (**C**) Scatter plot illustrating the logarithmic distribution of the normalized gRNA counts in the control (uninfected) and MHV-infected pools of cells for each individual *Smarca4* gRNA, as shown in panel B.

β-CoVs continue to pose a significant risk to global health. Identifying the host genes and cell death pathways implicated in β-CoV pathogenesis is critical for understanding the underlying mechanisms that drive pathogenesis and identifying treatment targets. Therefore, we evaluated a previous genome-wide cell death CRISPR knockout screen to identify potential host factors that regulate cell death during β-CoV infection [22]. This screen utilized the archetypal β-CoV MHV, along with iBMDMs derived from Cas9-transgenic mice and the mouse whole genome pooled Brie library (Addgene, 73633) [30]. The screen identified several host genes that may be involved in MHV-induced cell death, based on the enrichment of their respective gRNA counts (Figure 1A). Cells that are susceptible to MHV-mediated cell death are eliminated by infection during the CRISPR screen; therefore, cells in which critical cell death molecules have been deleted will be retained, resulting in a relative increase in the gRNA counts for these genes. To validate our analysis pipeline, we confirmed that we could detect the previously identified and characterized host gene *Ceacam1* as a top hit (Figure 1A). CEACAM1 is a known MHV receptor, and its deletion is known to inhibit MHV-mediated cell death [22]. Based on this validated pipeline, we leveraged this dataset and identified the enrichment of individual gRNAs that corresponded to the SWI/SNF-related, matrix-associated, actin-dependent regulator of chromatin, subfamily a, member 4 (SMARCA4) (Figure 1A). Furthermore, analysis of individual gRNAs demonstrated that all four distinct gRNAs targeting *Smarca4* were enriched in the MHV-infected pool of cells, although the specific level of enrichment varied (Figure 1B,C). Together, these results suggest that SMARCA4 plays a key role in MHV infection-induced cell death.

### 3.2. SMARCA4 Is a Regulator of MHV-Induced Inflammatory Cell Death

**Figure 2:**
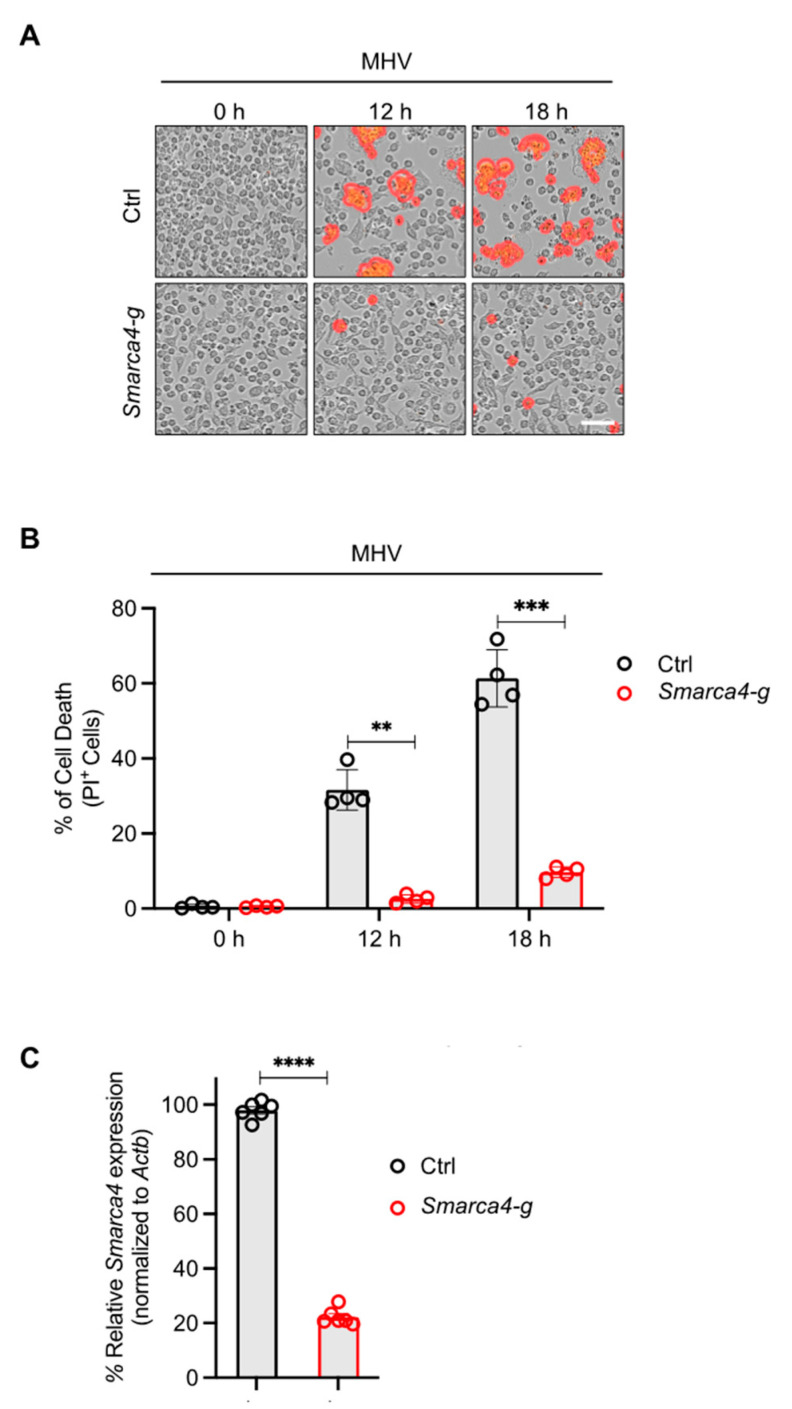
**SMARCA4 is required for MHV-induced inflammatory cell death**. (**A**) Representative images of cell death in mouse hepatitis virus (MHV; MOI 0.1)-infected immortalized bone marrow-derived macrophages (iBMDMs) with and without *Smarca4* gRNA treatment at the indicated time points. The red mask denotes dead cells, and the scale bar represents 50 μm. (**B**) Quantification of the percentage of cells undergoing lytic cell death at specified time points following MHV infection in iBMDMs treated with or without *Smarca4* gRNA. (**C**) *Smarca4* expression in uninfected iBMDMs treated with and without *Smarca4* gRNA. *Actb* was used to normalize *Smarca4* expression. The reported data are representative of two independent experiments with 4–6 technical replicates (**A**–**C**). Similar results were obtained for each experiment. The data are shown as the mean ± SEM (**B**,**C**). The Student’s *t*-test was used to determine statistical significance. ** *p* < 0.01; *** *p* < 0.001; and **** *p* < 0.0001. Ctrl: Control with no gRNA; *Smarca4-g*: *Smarca4* gRNA-treated.

The results of the CRISPR screen suggested a potential role for SMARCA4 in cell death in response to MHV infection (Figure 1). Therefore, we sought to confirm these CRISPR screen results through functional validation. We evaluated cell death kinetics in MHV-infected iBMDMs and observed a significant time-dependent increase in lytic cell death in wild-type (WT) iBMDMs infected with MHV (Figure 2A,B). Additionally, gRNA-mediated deletion of *Smarca4* inhibited MHV-induced cell death (Figure 2A–C).

**Figure 3:**
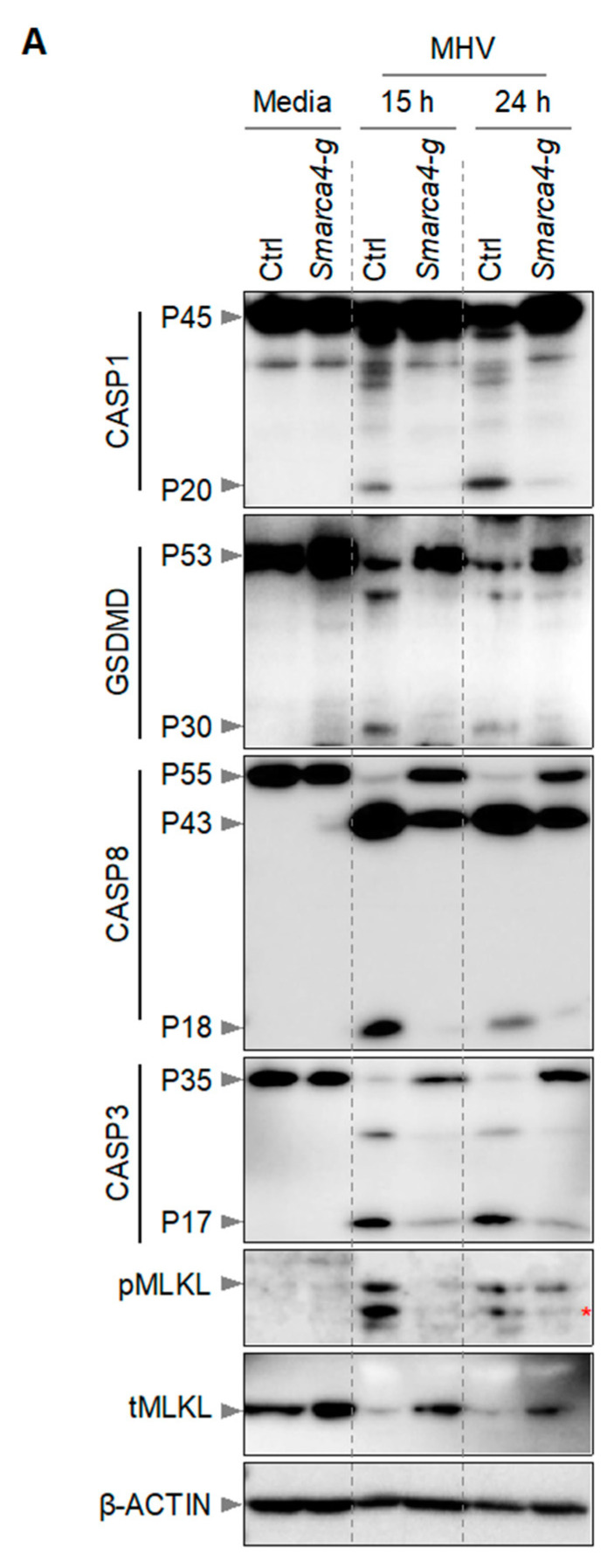
**SMARCA4 is required for MHV-induced PANoptosis**. (**A**) Immunoblot analysis of pro- (P45) and cleaved caspase-1 (P20; CASP1), pro- (P53) and activated (P30) gasdermin D (GSDMD), pro- (P55) and cleaved caspase-8 (P43, P18; CASP8), pro- (P35) and cleaved caspase-3 (P17; CASP3), and phospho-mixed lineage kinase domain-like pseudokinase (pMLKL) and total MLKL (tMLKL) from mouse hepatitis virus (MHV; MOI 0.1)-infected immortalized bone marrow-derived macrophages (iBMDMs) with and without *Smarca4* gRNA treatment at the indicated time points. Blots were reprobed using β-ACTIN antibody as an internal control. The reported data are representative of two independent experiments. Similar observations were obtained in each experiment. Ctrl: Control with no gRNA; *Smarca4-g*: *Smarca4* gRNA-treated. The red asterisk (*) denotes a non-specific band.

It has been previously reported that macrophages infected with MHV undergo PANoptosis, which involves the activation of multiple caspases, RIPKs, and other effectors and executioners of inflammatory cell death [2,12,22]. Therefore, we next investigated the effect of *Smarca4* loss on the activation of PANoptosis molecules in iBMDMs. Following MHV infection, we detected cleaved and active caspase-1 (indicating inflammasome activation) in WT iBMDMs (Figure 3). Caspase-1 cleaves GSDMD into its P30 N-terminal domain fragment (GSDMD-N), which promotes the formation of cell membrane pores and results in cell death [31,32,33,34,35]. Consistently, we also observed the P30 fragment of GSDMD in WT iBMDMs following MHV infection (Figure 3). In addition, we observed cleavage of caspases-8 and -3 in WT iBMDMs in response to MHV infection (Figure 3). However, gRNA-based deletion of *Smarca4* diminished the cleavage of these caspases and GSDMD. Furthermore, we observed phosphorylation of mixed lineage kinase domain-like pseudokinase (MLKL) in MHV-infected WT iBMDMs, which was reduced in *Smarca4*-depleted cells (Figure 3). Overall, the gRNA-mediated loss of *Smarca4* reduced the activation of caspases-1, -3, and -8, GSDMD, and pMLKL in response to MHV infection (Figure 3). These data collectively validated the role of SMARCA4 in MHV-induced PANoptotic cell death.

## 4. Discussion

The recognition of viruses or viral PAMPs via innate immune PRRs activates signaling mechanisms that result in inflammatory cell death and the release of proinflammatory cytokines. These innate immune mechanisms are the key first lines of defense against infections [1,2]. Furthermore, cell death pathways play a critical role in the host’s ability to limit the spread of viruses. While inflammatory cell death is beneficial for combating viral infections, dysregulated innate immune responses can aggravate inflammation and drive pathology, which can be detrimental to the host [8,9,12,36].

In recent years, β-CoVs, such as SARS-CoV-2, have posed significant challenges to human health. Growing evidence suggests an association between inflammatory cell death pathways and disease severity in SARS-CoV-2 infection [9,10,11,12,37,38]. In particular, the PANoptosis pathway is induced in viral infections, including β-CoVs and SARS-CoV-2 [9,10,11,12,21,22,36,39]. PANoptosis has also been linked to a wide range of infections, inflammatory diseases, immune disorders, and cancers [13,14,15,16,17,18,19,20,21,29,40,41,42]. Innate immune sensors (including ZBP1, RIPK1, AIM2, and NLRC5 and NLRP12) that drive the assembly of PANoptosome cell death complexes are known for their central role in the immune response to pathogens, PAMPs, DAMPs, and sterile triggers [18,20,21,39,43,44,45]. In the context of β-CoVs, ZBP1 plays a key role in mediating PANoptosis [12]. Furthermore, the cytokines TNF and IFN-γ serve as triggers for initiating PANoptosis and are known to exacerbate COVID-19 [9,10,46]. Neutralizing TNF and IFN-γ decreases mortality in SARS-CoV-2 murine models [9], indicating that cytokine-induced PANoptosis plays a critical role in disease pathogenesis.

Increasing evidence suggests that PANoptosis is a key driver of β-CoV infection severity and outcomes, particularly in the context of SARS-CoV-2 [9,10,11], and additional research is required to fully understand the upstream molecular mechanisms that initiate PANoptosis to identify new therapeutic targets. Our results identified *Smarca4* as a significantly enriched gRNA in a genome-wide CRISPR screen of host regulators of MHV-induced cell death, suggesting its role as a key upstream host factor in β-CoV-mediated PANoptosis. SMARCA4 is a critical regulator of chromatin organization and transcription [47,48]. RNA viruses are known to require host transcriptional machinery to enhance their replication [49], and it is possible that SMARCA4 is one of the key host factors required for β-CoV replication. SMARCA4 has also been previously identified as a top hit in other genome-wide CRISPR–Cas9-based screens and as an enriched molecule in several human cell lines and cell types in the context of SARS-CoV-2 viral infection [50,51,52]. However, in these previous studies, SMARCA4 was identified as a regulator of ACE2 expression [51], the key SARS-CoV-2 receptor [53,54,55]. As CEACAM1, and not ACE2, is the key receptor for MHV invasion [56], our results suggest that SMARCA4 acts through an alternative mechanism to regulate MHV-induced PANoptosis. However, future studies should address whether SMARCA4 regulates the expression of CEACAM1 or is required for viral replication during MHV infection in myeloid and non-myeloid cell types.

Overall, our findings substantiate and expand the importance of genome-wide screens for identifying host factors, such as SMARCA4, that regulate PANoptosis in response to β-CoV. These results contribute to our understanding of the role of the innate immune response in viral pathogenesis and cell death. Moreover, these findings suggest that targeting SMARCA4 may have therapeutic applications in limiting viral pathogenesis.

## Data Availability

Next-generation sequencing results from the CRISPR screen have been deposited in the BioProject: PRJNA1009133. All other datasets are included in the published article and the supplementary information.
